# Oxidative activity of corpus luteum and ovarian parenchyma in *Bos taurus indicus* heifers

**DOI:** 10.1590/1984-3143-AR2022-0084

**Published:** 2023-09-04

**Authors:** Suellen Miguez González, Marcela Bortoletto Cerezetti, Larissa Zamparone Bergamo, Camila Rodrigues Ferraz, Waldiceu Aparecido Verri, Marcelo Marcondes Seneda

**Affiliations:** 1 Laboratório de Biotecnologia da Reprodução Animal, Departamento de Clinicas Veterinárias - Centro de Ciências Agrarias, Universidade Estadual de Londrina, Londrina, PR, Brasil; 2 Departamento de Ciências Patológicas, Centro de Ciências Biológicas, Universidade Estadual de Londrina, Londrina, PR, Brasil

**Keywords:** bovine, free radicals, luteal tissue, ovarian tissue, ROS

## Abstract

The aim of this study was to evaluate the oxidative stress in ovaries and corpus luteum (CL) of *Bos taurus indicus* females and the oxidant effect of CL in ovarian tissues in regions near, intermediate, or distant from it. Ovaries (n=12) of Nelore heifers (n=6) were collected from a slaughterhouse and fragmented. Experiment 1, each ovary was obtained from three fragments, resulting in 18 fragments of ovaries with CL (OV+CL) and another 18 fragments of ovaries without CL (OV-CL). Three fragments were generated from CL, totaling 18 CL fragments. In experiment 2, the ovarian fragments were removed from specific regions near, intermediate, or distant from the CL. All the fragments were placed in *Eppendorf*-type microtubes (1 mL), kept in a thermal container at 4 ºC, and then stored in a -80 ºC freezer for analysis of oxidative stress (TBARS and NBT) and antioxidant potential (FRAP and ABTS). In the antioxidant activity analysis, luteal tissues showed more antioxidant activity than ovarian tissue (FRAP = P < 0.0001; ABTS = P < 0.02). In the oxidative stress analysis, CL had lower levels of reactive oxygen species (ROS; TBARS = P < 0.03; NBT = P < 0.0001) than ovarian tissues. There was no difference in antioxidant activity and oxidative stress between the fragments obtained from different regions (OV+CL *versus* OV-CL; P > 0.05). The presence of CL in the ovaries of *Bos taurus indicus* females did not influence the oxidative stress or antioxidant potential of the gonad. Thus, the removal of ovarian fragments with or without the presence of CL indicates that biotechnologies such as *in vitro* follicle cultivation is possible.

## Introduction

In mammalian species, the main function of the corpus luteum (CL) is in the synthesis of progesterone. This is necessary for the establishment of an adequate uterine environment to enhance the development of the peri-implantation and success in the progression and maintenance of pregnancy. Progesterone acts on the uterine endometrium to regulate the synthesis of growth factors, cytokines, adhesion proteins, protease inhibitors, hormones, and enzymes, which are the primary regulators of conceptus implantation, survival, and development (Graham and Clarke, 1997). However, inefficient production of progesterone by the CL is a potential risk factor for prenatal development and pregnancy (Arredondo and Noble, 2006; Diskin and Morris, 2008; Al-Gubory et al., 2012).

Periodic regression of CL causes the initiation of a new reproductive cycle. Thus, regression of the CL at each non-fertile cycle is characterized by the inability of the luteal cells to produce and secrete progesterone (functional regression) and cause the death of luteal cells (structural regression). Mammalian CL contains two types of steroidogenic cells, designated as small and large luteal cells (Al-Gubory et al., 2012). Although, the mechanisms of cell death and maintenance of progesterone production are complex and vary among mammalian species, it has been suggested that antioxidants play a significant role in CL during estrous cycle (Al-Gubory et al., 2005, 2006; Sugino, 2006; Amaral et al., 2021).

The production and propagation of reactive oxygen species (ROS) by CL depends on several regulatory factors, such as luteal antioxidants, steroid hormones, and cytokines (Al-Gubory et al., 2012; Amaral et al., 2021). However, the factor that has the greatest contribution to CL function is unknown. Furthermore, the sequence of events that leads to functional and structural luteal regression at the end of estrous cycle has not been fully elucidated.

*In vivo* studies on the CL of rats (Sugino et al., 1993), women (Sugino et al., 2000), sheep (Al-Gubory et al., 2004; Arianmanesh et al., 2011), and cows (Amaral et al., 2021) have shown the importance of antioxidant enzymes in controlling CL function during pre-implantation period. The presence of relevant amounts of antioxidants in CL has already been described in bovine and swine females, with emphases on β-carotene and ascorbic acid (Miszkiel et al., 1999; Al-Gubory et al., 2012). β-carotene can act on ovaries and uterus, protecting them against oxidative damage and ensuring a favorable environment for the development of follicles and embryos, respectively. The ascorbic acid content of mature CL is high in cattle, remains high during pregnancy, and decreases during CL regression (Petroff et al., 1997; Amaral et al., 2021).

ROS, such as O_2_, H_2_O_2_, and lipid peroxides, are generated by luteal cells during physiological regression of CL or induced by prostaglandin in mice, which may be associated with the reversible depletion of ascorbic acid present in these cells (Agarwal et al., 2003; Xu et al., 2021). In the last decade, the role of ROS in the reproduction of female mammals has been intensively studied, because high ROS production can interfere with the development of reproductive pathologies and infertility (Al-Gubory et al., 2012; Ávila et al., 2016; Wang et al., 2021).

The effect of ROS produced by CL on the ovary is still poorly understood. In bovine species, some biotechniques, such as the manipulation of oocytes in preantral ovarian follicles and IVEP, use follicles or oocytes from ovaries in the presence of CL. A study considering the presence in the CL at the time of the Ovum Pick Up suggested that it may interfere with oocyte viability, however, there was no ROS analysis (Hajarian et al., 2016). Thus, it is not known whether there is an influence of oxidative stress or antioxidant potential of CL in the ovary that encompasses it or whether the use of structures or ovarian tissue close to the CL could alter the outcome of these biotechnologies. Therefore, this study aimed to analyze the antioxidant potential and oxidative activity of the CL and ovaries of *Bos taurus indicus* females.

## Methods

### Research Ethical Committee

The ovaries used in this experiment were collected from slaughtered animals, destined for commercial slaughter, by following all rules established by Federal Law No. 1,283 which regulates the industrial and sanitary inspection of products of animal origin in Brazil. According to the standards of the Ethics Committee for Animal Experimentation of the State University of Londrina, there is no obligation of ethics evaluation when there are no interactions with the animals.

### Source of ovaries

Ovary samples (n=12) from cyclic heifers (n=6) of the Nellore breed were collected for the study. These females had a body condition score (BCS) between 3 and 4 (scale from 0 to 5; Ayres et al., 2009). All ovaries were subjected to oxidative stress analysis (ABTS - 2,2'-azinobis-3-ethylbenzothiazoline-6-sulfonic acid; FRAP - Ferric Reducing Antioxidant Power; TBARS - Thiobarbituric Acid Reative Substances; and NBT - Nitroblue Tetrazolium). The same pairs of ovaries were used for experiment 1 and 2. All procedures involving ovarian fragmentation were performed immediately after slaughter. After obtaining pairs of ovaries, they were selected according to the characteristics of the corpus luteum. Thus, the pair of ovaries that had a large corpus luteum, reddish color and a notable presence of blood vessels were chosen for the study.

To allow fragmentation of the ovary and CL, the adjacent tissues involving the ovaries were removed using forceps and a scalpel. Pairs of the ovaries were individually identified and washed in three saline baths (0.9%; JP Farma, Sao Paulo, Brazil). Subsequently, the ovaries were fragmented with the aid of a sterile disposable dermatological punch (6 mm; Kolplast, Sao Paulo, Brazil), resulting in fragments of approximately 9 mm^2^. One ovary of each pair that had CL were randomly included in the study.

#### Experiment 1

**Figure 1:**
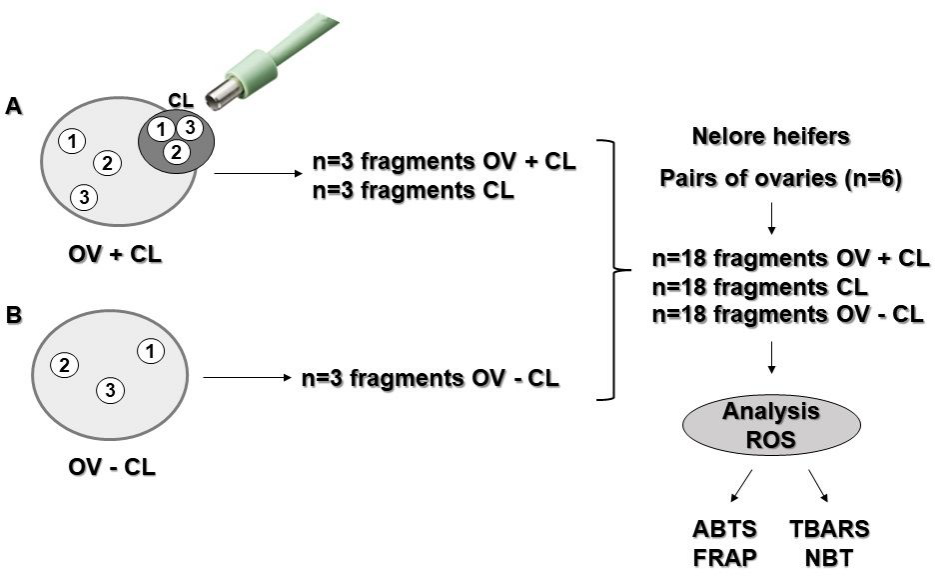
Demonstration scheme showing how fragments of pairs of ovaries and CL were obtained from *Bos taurus indicus* females. Ovary fragments with CL (Ovary with Corpus Luteum, OV+CL) and without CL (Ovary without Corpus Luteum, OV-CL; A and B, respectively) were collected. In addition to the antioxidant potential and oxidative stress analyses performed on the CL sections (2,2′-azino-bis-(3-ethylbenzothiazoline-6-sulfonic (ABTS) and Ferric reducing ability of plasma (FRAP); Thiobarbituric Acid Reactive Substances Assay (TBARS) and Nitroblue tetrazolium (NBT), in that order).

Three fragments were obtained from each ovary, totaling 36 fragments of six pairs of ovaries, of which 18 fragments were with CL (OV+CL) and the other 18 fragments were without CL (OV-CL). One ovary of each pair that was CL gave rise to three fragments, totaling 18 CL fragments (Figure 1). The proposed comparisons for the evaluation of ROS were as follows: ovary *versus* CL (OV *versus* CL), ovary with CL *versus* ovary without CL (OV+CL *versus* OV-CL), ovary with CL *versus* CL (OV+CL *versus* CL), and ovary without CL *versus* CL (OV-CL *versus* CL).

#### Experiment 2

**Figure 2:**
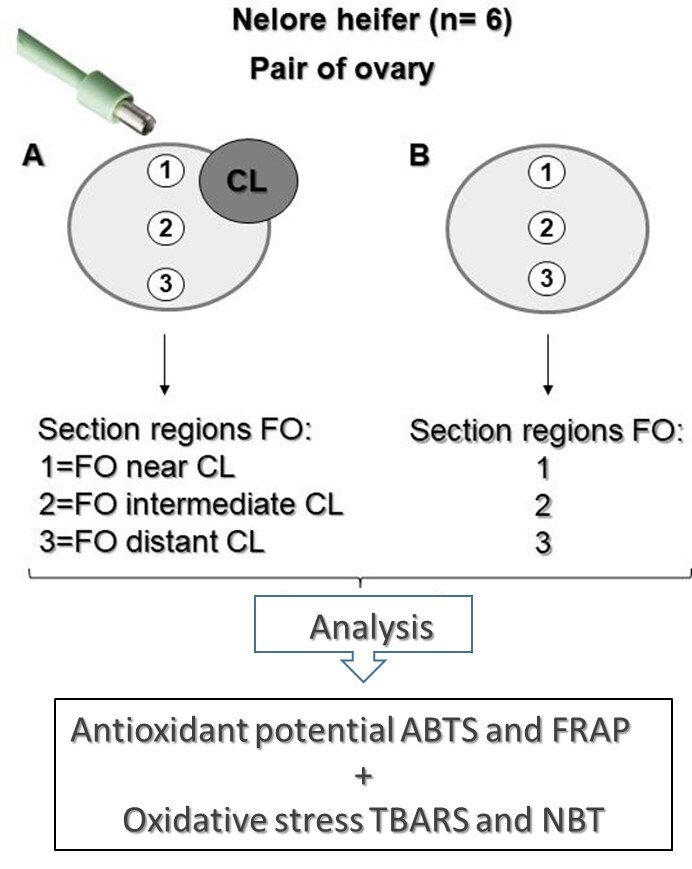
Demonstration scheme showing how ovarian fragments with or without CL (A and B, respectively) were collected from specific regions near, intermediate, and distant from the CL, named 1, 2, and 3, respectively for the analyses of antioxidant potential and oxidative stress. ABTS: 2,2′-azino-bis-(3-ethylbenzothiazoline-6-sulfonic; FRAP: Ferric reducing ability of plasma; TBARS: Thiobarbituric Acid Reactive Substances Assay; NBT: Nitroblue tetrazolium; FO - fragments of ovary.

Each ovary was sectioned into three fragments, totaling 36 fragments from six pairs of ovaries. These three fragments were taken from specific regions of the ovary near, intermediate, or distant from the CL, called regions 1, 2, and 3, respectively (Figure 2). To determine regions 1, 2 and 3, in ovaries with and without CL, we drew a vertical line in the middle region of the ovary and thus fragment 1 was removed close to the greater curvature of the ovary (consequently closer to the corpus luteum, since this structure forms most frequently on the greater curvature of the ovary). Fragment 2 was removed from the middle region of the ovary (just below fragment 1 - intermediate region) and fragment 3 was removed close to the ovarian pedicle (consequently further away from the corpus luteum). The comparisons proposed for the analysis of ROS were ovaries with CL *versus* ovaries without CL (OV+CL *versus* OV-CL), based on each region of ovarian fragment removed, that is, region 1, 2, and 3 (near, intermediate, and distant from the CL).

### Analyses of oxidative stress and antioxidant activity

The fragments were placed individually in Eppendorf-type microtubes (1 mL), kept in a thermal container (4°C) for transport from the slaughterhouse to the laboratory, where they were immediately stored in a freezer at -80°C for subsequent analysis oxidative and antioxidant. Samples were processed and analyzed after five days of storage. Ovarian and CL fragments were subjected to oxidative stress levels analysis using ABTS and FRAP kinetic-colorimetric assays to determine the ability of ovarian tissue to resist oxidative damage. TBARS and NBT, through the determination of lipid peroxidation and production of superoxide anions, indicate oxidative stress in the samples. The assays were performed as previously described by Pinho-Ribeiro et al. (2016).

### Antioxidant activity analysis - using ABTS and FRAP assay

Samples of the ovarian fragments were ground, homogenized with 500 µL of 1.15% KCl, and centrifuged (10 min × 1500 rpm × 4 ºC). “Analyses regarding the antioxidant potential assessed FRAP (ability to reduce iron) and ABTS (its ability to neutralize free radicals) (Re et al., 1999; Katalinic et al., 2005). To measure FRAP, 10 µL of the supernatant was mixed with 40 µL of deionized water and 150 µL of the FRAP reagent; the absorbance was measured at 595 nm. For ABTS evaluation, 200 µL of diluted ABTS solution was mixed with 20 µL of the supernatant, and the absorbance was measured at 730 nm after 6 mins. The results are presented as nmol equivalents of 6-hydroxy-2,5,7,8-tetramethylchroman-2-carboxylic acid (TROLOX) per mg of protein.

### Oxidative stress analysis

#### Lipid peroxidation using the TBARS assay

Thiobarbituric acid reactive substances (TBARS) assay was performed based on the levels of malondialdehyde (MDA), an intermediate product of lipid peroxidation. To carry out this test, the homogenate (50 μL) obtained previously in the FRAP test was placed in an *Eppendorf*-type microtube (1 mL) with iron chloride (FeCl_3_; 5 μL), ascorbic acid (5 μL), trichloroacetic acid (TCA; 50 μL; 2.8%), and thiobarbituric acid (TBA; 50 μL; 1.0%).

Thereafter, the mixture was shaken and maintained in a water bath (90 ºC) for 15 min. Subsequently, the samples were cooled in an ice bath, shaken again for 15 min (4º C), and centrifuged (3000 rpm). The supernatant contained in the microtubes was distributed in 96-well plates, and MDA levels were determined by the difference between the absorbance at 535 and 572 nm using a spectrophotometer and microplate reader. Results of the TBARS assay are presented as optical density (OD) per mg of protein.

#### Superoxide anion production using the NBT assay

The nitroblue tetrazolium (NBT) assay was used to measure the production of superoxide anions, and NBT reduction was measured using a microplate reader spectrophotometer at 600 nm (Multiskan GO, Thermo Scientific). The homogenate (50 μL) was added to NBT (100 μL, 1 mg/mL) in 96-well plates. After the incubation period (15 min), all samples were discarded from the plate, and potassium hydroxide (KOH; 120 μL) and dimethyl sulfoxide (DMSO; 120 μL) were then added. The results are presented as OD per mg of protein.

### Statistical analysis

The analyses of the oxidative stress (TBARS and NBT) and antioxidant activity (ABTS and FRAP) of the ovarian and CL fragments (experiment 1) in each ovarian region studied in experiment 2 (regions near, intermediate, or distant from the CL) were performed, and the results were expressed in mg of protein. Comparisons of these measurements were performed using t-test. All analyses were performed using GraphPad Prism 8.0, and a 5% significance level was adopted.

## Results

### Experiment 1

**Figure 3:**
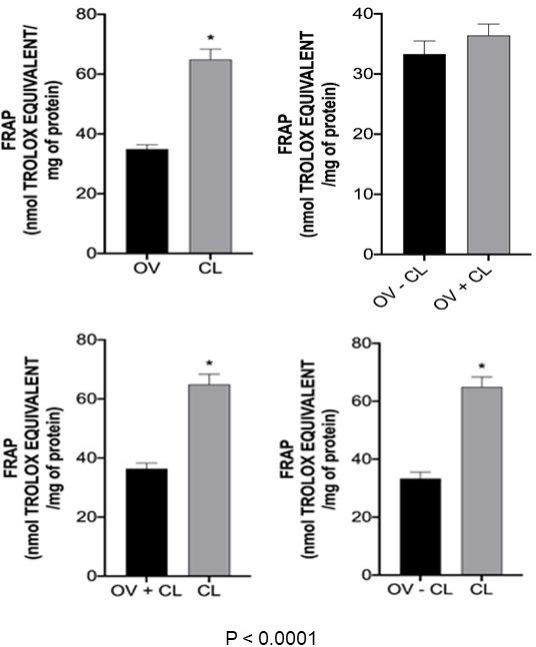
FRAP analysis of OV *versus* CL, OV-CL *versus* OV+CL, and OV+CL *versus* CL comparisons. The asterisk over the chart columns indicates a significant difference (P ≤ 0.05). OV: ovary; CL: Corpus Luteum; FRAP: Ferric reducing ability of plasma.

**Figure 4:**
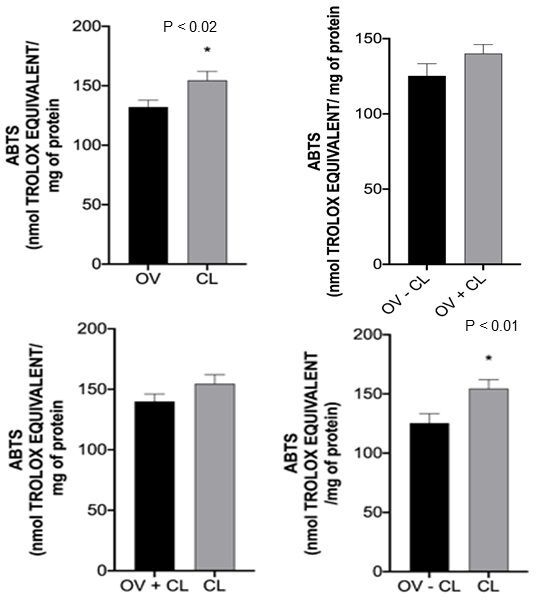
ABTS analysis of OV *versus* CL, OV-CL *versus* OV+CL, and OV+CL *versus* CL comparisons. The asterisk over the chart columns indicates a significant difference (P ≤ 0.05). OV: ovary; CL: Corpus Luteum; ABTS: 2,2′-azino-bis-(3-ethylbenzothiazoline-6-sulfonic.

With regard to the analysis of antioxidant potential, FRAP (iron reduction ability) in OV *versus* CL and OV+CL *versus* CL comparisons indicated that luteal tissues have higher antioxidant activity (P < 0.0001; Figure 3) than ovarian tissues. In addition, the ABTS (free radical neutralization ability) in the OV *versus* CL and OV-CL *versus* CL comparisons indicated that luteal tissues have higher antioxidant activity (P < 0.02 and P < 0.01, respectively; Figure 4) than ovarian tissue.

**Figure 5:**
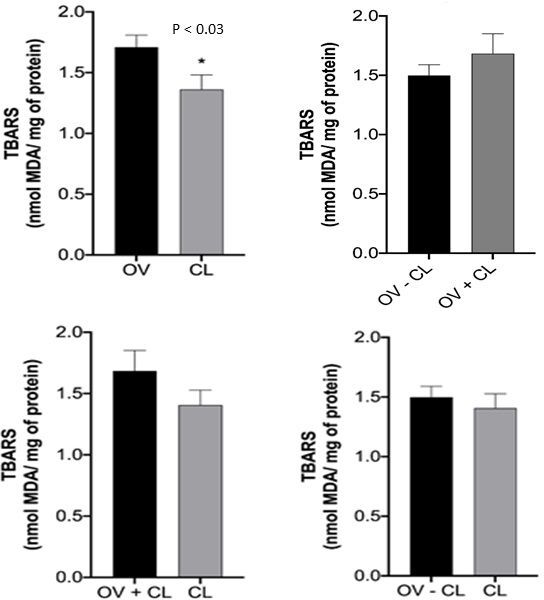
TBARS analysis of OV *versus* CL, OV-CL *versus* OV+CL, and OV+CL *versus* CL comparisons. The asterisk over the chart columns indicates a significant difference (P ≤ 0.05). OV: ovary; CL: Corpus Luteum; TBARS: Thiobarbituric Acid Reactive Substances Assay.

**Figure 6:**
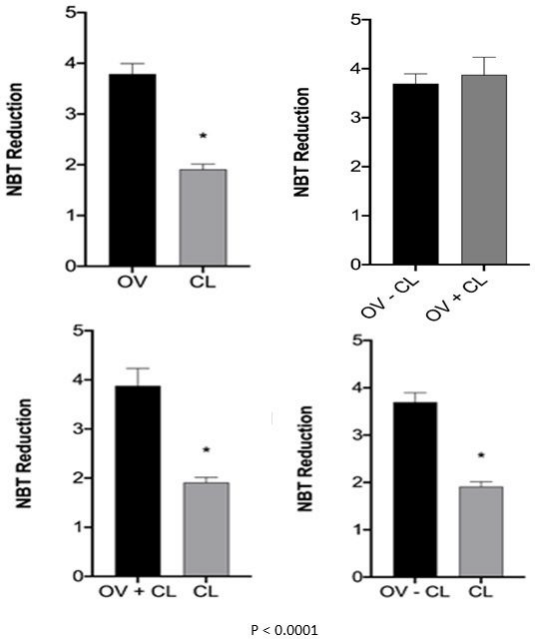
NBT analysis of OV *versus* CL, OV-CL *versus* OV+CL, and OV+CL *versus* CL comparisons. The asterisk over the chart columns indicates a significant difference (P ≤ 0.05). OV: ovary; CL: Corpus Luteum; NBT: Nitroblue tetrazolium.

In oxidative stress analysis, TBARS (lipid peroxidation) in the OV *versus* CL comparisons indicated that luteal tissues experienced lower oxidative stress than the ovarian tissues (P < 0.03; Figure 5). The superoxide anion production (NBT) in the OV *versus* CL and OV-CL *versus* CL comparisons also indicated that the luteal tissue experienced lower oxidative stress than the ovarian tissues (P < 0.0001; Figure 6).

### Experiment 2

In this study, each fragment obtained from regions 1, 2, and 3 (near, intermediate, and distant from the CL, respectively) was compared with the sectioned ovarian fragments without CL from the same regions (OV+CL *versus* OV-CL). FRAP, ABTS, TBARS, and NBT analyses did not indicate significant differences in antioxidant activity and oxidative stress between the regions (OV+CL *versus* OV-CL; P > 0.05).

## Discussion

The present study showed that bovine luteal tissue has greater antioxidant activity and less oxidative stress than ovarian tissue. In addition, the presence of CL in the ovary of *Bos taurus indicus* females, regardless of the region (near, intermediate, or distant from the CL) of removal of the ovarian fragment, did not result in significant differences in the analyses of antioxidant potential and oxidative stress. These results may be related to the physiological characteristics of CL, which is a transient gland in the ovary. Some studies have indicated that luteal tissue has specific mechanisms against ROS, acting to preserve their steroidogenic activity (Kato et al., 1997; Sugino, 2006; Xu et al., 2021). Other studies have suggested that there is a balance between the increase in luteal steroidogenic activity and the antioxidant effects induced by high concentrations of P4. The interaction between these events leads to increased availability of antioxidants, such as ascorbate and carotenoids, thus preserving this transient gland (Peltier et al., 2006; Jones et al., 2008; Al-Gubory et al., 2012). The greater antioxidant activity found in the luteal tissue than the ovarian tissue may be related to its own mechanisms, which guarantee the viability of its endocrine activity, and may have substances with antioxidant functions.

However, the antioxidant potential and oxidative stress in the ovary and CL of bovine species are unknown. The processes involved in carrying out these analyses are considered laborious, because all the fragments must be processed at the same period to obtain a reliable reading of the tissues. Thus, there are few studies covering the antioxidant activity and oxidative stress in ovarian and luteal tissues in females. In canine species, the concentration of ROS in their luteal phase decreased compared to the concentrations of ROS during the follicular phase (Rizzo et al., 2009). A survey of polyestrous dairy cows showed high concentrations of ROS and low concentrations of P4 during the critical period of CL survival, resulting in failure to conceive (Rizzo et al., 2007). This is possible because stressors can lead to an overproduction of ROS, which hinders the functionality of luteal cells, such as the synthesis of P4 (Rizzo et al., 2007; Al-Gubory et al., 2012). Wang et al. (2019) studied the effect of heat stress on the *in vitro* culture of bovine granulosa cells and showed that there are deleterious effects that induce apoptosis. In this study, the cytoprotective mechanism of OH was characterized to elucidate the activity of enzymes and oxidative stress markers.

In human medicine, the deleterious effects of oxidative stress on ovarian tissue are the development of injuries and infertility (Fang et al., 2019; Jin et al., 2019; Wang et al., 2021). The drug with the greatest potential for toxicity is the chemotherapy drug doxorubicin (DOX), used to treat breast cancer in women. The administration of such active ingredient causes oxidative stress to the ovaries, activating the production of ROS and interfering with the vital function of the mitochondria. So the study emphasizes that nuclear factor erythroid 2 - related factor 2 (Nrf2) acts in the ovaries. Nrf2 is considered to be the master regulator of the body's antioxidant response and is an important protein for tissue homeostasis (Niringiyumukiza et al., 2019). Ourstudy analyzed the antioxidant activity and oxidative stress of bovine ovarian tissue; and the result showed that the antioxidant activity of ovarian tissue is lower than that of luteal tissues. Thus, we suggest that the regulatory factor Nrf2 may be involve in the response to oxidative stress in the bovine ovary, allowing a balanced environment for ROS and enabling the growth and regression of follicles repeatedly during the reproductive life of the female.

This study also evaluated the influence of the presence of CL on the ovary using ovarian fragments collected from three distinct regions (near, intermediate, and distant from the CL). Comparison results showed that there was no significant difference in antioxidant potential and oxidative stress between the regions and their respective ovarian fragments, and the reason is unknown, suggesting that the inclusion of CL in the ovary does not influence the environment or the balance of ROS. Sugino et al. (2004) and Amaral et al. (2021) reported the involvement of ROS in folliculogenesis and ovarian steroidogenesis in follicular fluid. Granulosa cell hypoxia induces follicular angiogenesis, an important interaction that promotes follicular growth and development. Angiogenesis occurs in granulosa and theca cells, with greater intensity after the LH. Thus, the impairment of angiogenesis within the ovary, is considered one of the mechanisms through which follicular atresia occurs (Tropea et al., 2006; Xu et al., 2021). The participation of ROS as a pathway of follicular atresia suggests that the detection of higher levels of oxidative stress in the ovarian tissue than in the luteal tissue may be plausible.

Research related to biotechnologies such as *in vitro* culture of bovine preantral follicles have shown the use of different substances in culture medium to mimic the physiological environment of the ovary. Recent studies have included antioxidant substances such as alpha-lipoic acid and quercetin at different concentrations in the *in vitro* culture media of preantral follicles included in bovine ovarian fragments (Bergamo et al., 2022; Cerezetti et al., 2021). The results are promising because the fragmentation of the ovary and changes in the environment could generate ROS and restrict the success of *in vitro* growth of preantral follicles. The present study analyzed the antioxidant activity and oxidative stress of the ovaries and CL of bovines, indicating the ambience found in fragments not cultured *in vitro*. Thus, this study established a reference for the analysis of ROS, on which biotechnologies such as *in vitro* culture of preantral follicles could be based and compared. Another questionable aspect that has been clarified is the influence of ROS from CL on ovarian fragments close or not close to it. Thus, the removal of ovarian fragments close or not to the CL for experiments was avoided due to the scarcity of information about the influence of the CL on the ovarian tissue and how much it could affect the results of this environment. Our study demonstrated that the presence of CL did not have any significant influence on ROS production in bovine ovarian fragments, indicating that ovaries with CL can be used for *in vitro* cultivation experiments.

## Conclusion

This study highlights the antioxidant potential and oxidative stress in ovarian and luteal tissues in bovine species. The presence of CL in the ovaries of *Bos taurus indicus* females did not influence the oxidative stress or antioxidant potential of the gonad. The influence of CL on ovarian tissue indicates that the removal of ovarian fragments with CL is possible and can be successfully used in biotechnologies such as *in vitro* follicle culture.
